# Mechanical Modulation of Phonon-Assisted Field Emission in a Silicon Nanomembrane Detector for Time-of-Flight Mass Spectrometry

**DOI:** 10.3390/s16020200

**Published:** 2016-02-05

**Authors:** Jonghoo Park, Robert H. Blick

**Affiliations:** 1 Department of Electrical Engineering, Kyungpook National University, Daegu, 702-701, Korea; 2 Center for Hybrid Nanostructures (CHYN), Institute of Nanostructure and Solid State Physics, University of Hamburg, Jungiussstr. 11, 20355 Hamburg, Germany

**Keywords:** NEMS, MALDI TOF, silicon nanomembrane, mass spectrometry, phonon, ion detector

## Abstract

We demonstrate mechanical modulation of phonon-assisted field emission in a free-standing silicon nanomembrane detector for time-of-flight mass spectrometry of proteins. The impacts of ion bombardment on the silicon nanomembrane have been explored in both mechanical and electrical points of view. Locally elevated lattice temperature in the silicon nanomembrane, resulting from the transduction of ion kinetic energy into thermal energy through the ion bombardment, induces not only phonon-assisted field emission but also a mechanical vibration in the silicon nanomembrane. The coupling of these mechanical and electrical phenomenon leads to mechanical modulation of phonon-assisted field emission. The thermal energy relaxation through mechanical vibration in addition to the lateral heat conduction and field emission in the silicon nanomembrane offers effective cooling of the nanomembrane, thereby allowing high resolution mass analysis.

## 1. Introduction

Nanomembranes (NMs) are freestanding structures with thicknesses of on the order of a few hundred nanometers or less with aspect ratio over 1,000,000. The macroscopic mechanical degrees of freedom distinguishes NMs from bulk materials and it makes NMs versatile platforms for both fundamental and applied researches in the field of microelectromechanical [1,2,3,4,5,6] and optomechanical [7,8,9] systems. The silicon nanomembrane, thinner than its phonon mean free path (~300 nm at room temperature), has attracted considerable attention because it yields 2D phonon confinement and ballistic phonon transport which can offer opportunities for manipulating thermal transport [10,11].

Recently, we demonstrated quasi-diffusive phonon transport in the silicon nanomembrane for ion detection in matrix-assisted laser desorption/ionization time-of-flight (MALDI TOF) mass spectrometry of proteins [12,13]. In MALDI TOF mass spectrometry, ions are generated by MALDI process and accelerated in an electric field. The accelerated ions are injected into the field-free time-of-flight tube where they separate by mass-to-charge ratio (*m*/*z*) and reach a detector with different velocities according to their *m*/*z*. The sensitivity of the conventional detectors such as microchannel plate (MCP) detectors are highly dependent on the impact velocity of the ion. Since in TOF, the heavier ion fly more slowly than the lighter one, this results in severe decrease in sensitivity for the detection of large ion. However, in TOF, kinetic energy of the ions is same regardless of their masses, since they all are accelerated in the same electric field. Therefore, a measure of the amount of kinetic energy deposited in the detector by ion bombardment is highly independent on the ion mass and thus can overcome the drawbacks of the conventional ion detectors which stems from the velocity dependent sensitivity. The impact energy measurement in TOF mass spectrometry has been realized by the cryogenic detectors [14]. This approach demonstrated a quantum efficiency several orders of magnitude larger than the MCP detectors, but it requires an expensive cryogenic system. It has been known that when accelerated ions bombard the detector, roughly half of the ion’s kinetic energy is transformed into thermal energy [14]. In MALDI TOF, ions are usually accelerated by 20~25 kV, corresponding to the thermal energy of 10~12.5 keV.

In our earlier work, we utilized silicon nanomembrane thinner than its phonon mean free path to transform the thermal energy deposited by ion bombardment into the phonon-assisted field emission (PAFE) for ion detection in MALDI TOF mass spectrometry at room temperature. The detector exhibits exceptional mass range and high sensitivity for the detection of large ion up to ~1 MDa which is challenging to achieve using conventional detectors [12].

Here we demonstrate the mechanical modulation of phonon-assisted field emission in free-standing silicon nanomembrane. We have found that when the accelerated ions bombard the one side of the silicon nanomembrane and deposit thermal energy on the nanomembrane, it causes not only PAFE but also mechanical vibration of the silicon nanomembrane. The coupling of these electrical and mechanical phenomenon leads to mechanical modulation of PAFE and it gives better understanding on the operating principle of the detector and how thermal energy dissipates in the silicon nanomembrane detector.

## 2. Experimental Section

**Figure 1:**
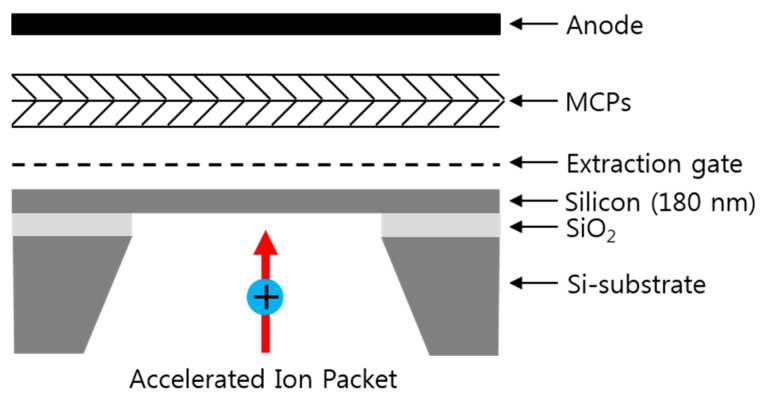
Schematic of the detector, consisting of a 180 nm silicon nanomembrane, an extraction gate, microchannel plates (MCPs), and an anode. Accelerated ions bombard the back side of the nanomembrane and deposit thermal energy on it.

The measurement set up for the mechanical modulation of the PAFE is graphically illustrated in the Figure 1. The detector consists of the 180-nm-thick, free-standing square silicon nanomembrane with a lateral dimension of 2 mm, an extraction gate, microchannel plates (MCPs), and an anode. The detector is place at the end of the MALDI TOF mass spectrometer (Voyager-DE STR, Perseptive Biosystems, Framingham, MA, USA). The fabrication of the free-standing silicon nanomembrane started from a SOI wafer. The device layer of the SOI wafer was thinned down to 180 nm by dry thermal oxidation. The substrate under the membrane area is etched by potassium hydroxide (KOH) solution using silicon nitride etch mask. The extraction gate consists of metal grid (70 lines-per-inch, line width of 18.5 μm) and is placed 127 μm above the silicon nanomembrane. The electric field is applied between the silicon nanomembrane and the extraction gate. When accelerated ions bombard the silicon nanomembrane, roughly half of the ion’s kinetic energy is transformed into the thermal energy [14]. In the silicon nanomembrane, thinner than its phonon mean free path, thermal energy deposited on one side by ion bombardment can be delivered to the other side very rapidly (faster than normal diffusion) through quasi-diffusive phonons transport. The phonons that reach the other side scatter diffusively at the surface due to the surface roughness. It consequently causes local heating and increasing the lattice temperature, thereby causing an increase in electron emission with assistance of electric field which thins and lowers the vacuum barrier. The number of electrons emitted from the silicon surface with help of thermal energy and electric field is amplified by the MCPs and collected by the anode. The collected current is measured by oscilloscope with internal resistance set to 1 MΩ.

## 3. Results and Discussion

**Figure 2:**
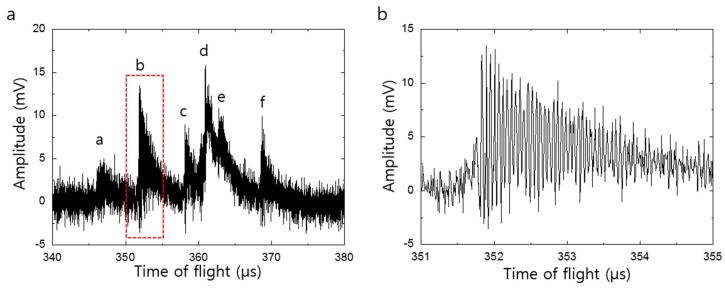
MALDI TOF mass spectrum for IgG. (**a**) Mass spectrum obtained using silicon nanomembrane detector after eliminating system noises by the band block filtering with the lower and upper cutoff frequency of 1 MHz and 8 MHz, respectively. It shows multiple peaks at the time-of-flight of the singly charged IgG; (**b**) The magnified view of the peak enclosed by dashed lines. This clearly shows modulation of phonon-assisted field emission which stems from the mechanical vibration of the nanomembrane with resonance frequency of 17.5 MHz.

Figure 2a shows the MALDI TOF mass spectrum for human immunoglobulin G (IgG, ~150,000 Da, 1 pmol/μL, Sigma Aldrich, St. Louis, MO, USA) in a sinapinic acid matrix. The IgG ions were accelerated through a potential of 23.5 kV and the voltage applied between silicon nanomembrane and the extraction gate was 1.5 kV. The applied electric field to the nanoemembrane is not sufficient to induce Fowler-Nordheim field emission [15] but it can yield Schottky emission [16,17] with assistance of thermal energy deposited by the ion bombardment. The MALDI TOF mass spectrum was acquired by averaging for 10 laser shots, followed by eliminating system noises by the band block filtering with the lower and upper cutoff frequency of 1 MHz and 8 MHz, respectively. The cutoff frequencies for the band block filtering were determined by the control experiment performed with same condition but without ion bombardment. The mass spectrum reveals multiple peaks and the time-of-flight of each peak corresponds to the *m*/*z* of 135.9 (a), 139.7 (b), 144.8 (c), 147 (d), 148.6 (e), and 153.4 kDa (f). Figure 2b shows a magnified view of the peak enclosed by dashed lines in Figure 2a. It shows that the amplitude of the peak is modulated by high frequency signal with oscillation frequency near 17.5 MHz.

**Figure 3:**
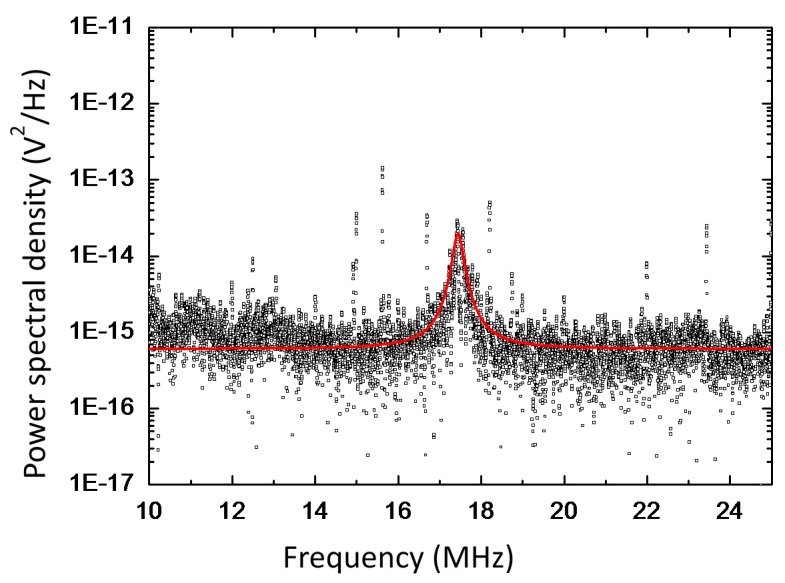
Power spectral density of the mass spectrum and its Lorenztian fit (red line), it clearly shows the resonance at 17.5 MHz. This resonance peak appeals only when the ions bombard the nanomembane.

Figure 3 shows the power spectral density of the MALDI spectrum in the frequencies above 10 MHz. The peak at the frequency of 17.5 MHz is revealed and it appears only when ions bombard the nanomembrane. The red line in Figure 3 is the Lorenztian fit of the spectrum. The power spectral density in the frequencies below 1 MHz is dominated by 1/*f*^2^ noise. No peak which associated with ion bombardment and has higher power spectral density than that of noise was found in the blocked frequency range.

**Figure 4:**
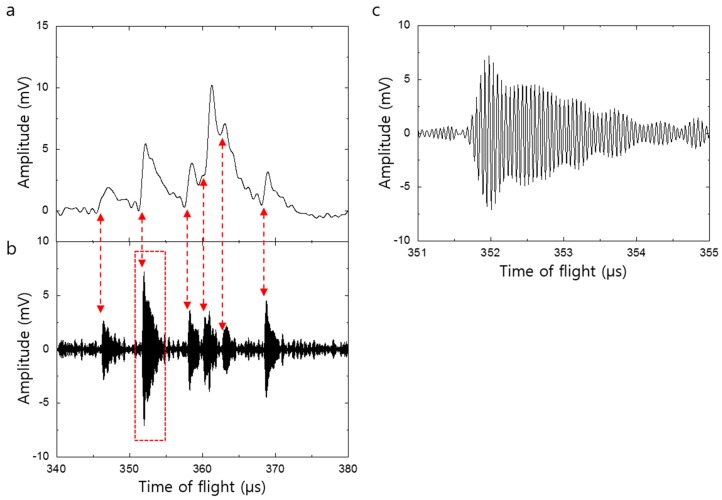
(**a**) Low frequency decomposition of Figure 2a, showing phonon-assisted field emission; (**b**) High frequency decomposition of Figure 2a, showing the mechanical vibration of the nanomembrane. The onset times of the mechanical oscillations coincide with the time-of-flight of the leading edge of the ion packet; (**c**) Magnified view of the peak enclosed by dashed lines in (**b**), clearly showing mechanical vibrations.

Figure 4a,b shows the decomposition of the mass spectrum shown in Figure 2a into the low and high frequency spectrum. The low frequency spectrum was extracted by low pass filtering with the cutoff frequency of 1 MHz. The high frequency spectrum was extracted by band pass filtering with the lower and upper cutoff frequency of 15 MHz and 20 MHz, respectively. The peaks in the high frequency spectrum arise from the mechanical vibration of the silicon nanomembrane while the peaks in low frequency spectrum stems from the PAFE. The time at which mechanical vibration begins coincides with the time at the peaks appeared in PAFE. The coupling of these electrical (low frequency spectrum) and mechanical (high frequency spectrum) phenomenon leads to the mechanical modulation of the PAFE and is clearly shown in Figure 2a. Figure 4c is a magnified view of the peak enclosed by dashed lines in Figure 4b and clearly shows a mechanical vibration with oscillation frequency of 17.5 MHz. The coupling mechanism can be described as follows. When accelerated ion bombard the nanomembrane, the kinetic energy of the ions is transformed into thermal energy and elevating temperature at the impact site. The thermal energy deposited on back side of the nanomembrane is delivered to the front side, thereby assisting the field emission. This process is well approximated by the expression [18]:

(1) $$J(T,V)=\frac{mek_{B}}{2\pi \hbar^{3}}T^{2}e^{-(W-\Delta W)/k_{B}T}$$

where *T* is the temperature, *V* is the voltage between the silicon nanomembrane and the extraction gate, *e* and *m* are the electron’s charge and mass, respectively. *W* is the work function of the silicon, and Δ*W* = (*eV*/4*πε*_0_*d*)^1/2^ is the field-dependent correction to the work function due to tunneling through the barrier with *d* being the distance between the silicon nanomembrane and the extraction gate. The dominant feature of this PAFE process is formed by the fast rise time and slow decay. The field emission increases rapidly as heat is transferred across the nanomembrane (out-of-plane direction) by quasi-diffusive phonon transport. Subsequently, the heat is spread laterally away from the field emission site by heat diffusion, which is much slower process than quasi-diffusive phonon transport. This process slowly lowers the lattice temperature and finally shuts off the field emission as shown in Figure 4a.

In the meantime, thermomechanical forces arose from the non-uniform temperature distribution across the nanomembrane induces mechanical vibrations. The vibrations of the nanomembrane due to the thermal force and the applied DC voltage onto the nanomembrane can be expressed by [19]:

(2) $$\rho h\ddot{\zeta }+D\Delta^{2}\zeta -F\Delta \zeta =\frac{-E\alpha }{3(1-2\sigma )}\nabla T-\frac{1}{2}V^{2}\frac{dC}{d\zeta }$$

where *ζ* is the vertical displacement, *ρ* and *h* are the density and the thickness of the nanomembrane, respectively. *F* is the stretching force per unit length of the edge of the nanomembrane, *E* is Young’s modulus, *α* is the thermal expansion coefficient, *σ* is the Poisson ratio, *T* is the temperature, *V* and *C* are the voltage and capacitance between the nanomembrane and the extraction gate, respectively, and *D* = *E*h^3^/12(1 − σ^2^) is the flexure rigidity. The mechanical vibrations alter the distance between silicon nanomembrane and the extraction gate, resulting in modulating of the field dependent work function, ΔW in Equation (1), thereby modulating PAFE. The mechanical vibration eventually relaxes back to thermal equilibrium as the nonuniform temperature distribution becomes uniform by heat conduction.

The silicon nanomembrane should be cooled before subsequent ion bombardment deposits additional thermal energy onto it in order to provide necessary resolution to separate any successive ion packets. However, the in-plane thermal conductivity of silicon nanomembrane decreases considerably as the thickness decreases by phonon-boundary scattering [20,21]. The PAFE provides an additional cooling mechanism through field emission. When the electrons are escaping from the silicon surface, they carry an energy amount at least equal to the work function of silicon, and it is known as Nottingham effect [22,23,24]. Our numerical calculations of the coupling heat diffusion and field emission process showed that the temperature reaches a maximum of only 770 K due to the spontaneous cooling from the field emission [12]. The electrons which are excited by lattice temperature higher than 770 K will escape from the surface carrying access amount of energy. Furthermore, the transduction of thermal energy into mechanical vibration also provides additional cooling mechanism to the nanomembrane, thereby allowing high resolution mass analysis in TOF mass spectrometry.

**Figure 5:**
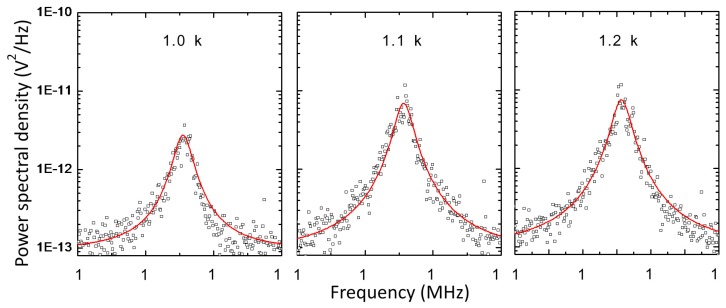
Power spectral density of mass spectrum for insulin under different voltages between the nanomembrane and the extraction gate. Red lines show Lorenztian fit of the power spectral density. As DC voltage increases, the power spectral density increases because it thins and lowers the vacuum barrier more. The resonance frequency of mechanical vibration is independent on the voltage between the nanomembrane and the extraction gate.

Figure 5 shows the power spectral density of the MALDI spectrum for insulin (5729 Da, 10 pmol/μL, Sigma Aldrich, St. Louis, MO, USA) in a sinapinic acid matrix for three different DC voltages (1.0, 1.1, and 1.2 kV) between the extraction gate and silicon nanomembrane. Each spectrum was obtained by averaging 10 power spectral density and red line is the Lorenztian fit of the spectrum. For low DC voltages (~0.6 kV), neither PAFE nor mechanical vibration has been revealed. This indicates that the elevated temperature in silicon nanomembrane after ion bombardment is not high enough to induce thermionic emission, although the kinetic energy of ions (23.5 keV) is extremely higher than the work function of the silicon (4.6~4.85 eV). It also indicates that the applied DC voltage is not high enough to sufficiently lower and thin the potential barrier so for electrons to tunnel through the barrier. As DC voltage increases, the power spectral density for the peak near 17.5 MHz increases. The increased DC voltage further lowers and thins the vacuum barrier, thereby allowing more electrons to overcome and tunnel through the vacuum barrier. It was also found that the resonance frequency of mechanical vibration was independent on DC voltage.

## 4. Conclusions

In summary, we have explored the mechanical modulation of the PAFE in silicon nanomembrane detector for time-of-flight mass spectrometry of proteins. The transduction of ion kinetic energy into the thermal energy gives rise to the mechanical vibration and PAFE in silicon nanomembrane. Each process provides additional cooling mechanism to the system, resulting in significant cooling of the nanomembrane compared to the conventional heat conduction, and consequently allowing high resolution mass analysis in MALDI TOF mass spectrometry.
